# Self-determined motivation in rehabilitating professional rugby union players

**DOI:** 10.1186/s13102-016-0065-6

**Published:** 2017-01-18

**Authors:** Fraser Carson, Remco C. J. Polman

**Affiliations:** 1 Centre for Sport Research, Deakin University, Melbourne, Australia; 2 Department of Psychology, Bournemouth University, Bournemouth, UK

**Keywords:** Autonomy, Competence, Relatedness, ACL injury

## Abstract

**Background:**

The aim of the present study was to explore the views of professional rugby union players during the early rehabilitation, late rehabilitation and return to play stages, following anterior cruciate ligament (ACL) injury.

**Methods:**

A qualitative dominant, mixed methodological approach was utilized with five players who had suffered an ACL injury requiring reconstructive surgery. A longitudinal approach, concurrent with each player’s rehabilitation, consisting of twice monthly interviews, a self-report diary and three established questionnaires (MOS-Social Support Survey, Sherbourne & Stewart, 1991; Sport Climate Questionnaire, Deci & Ryan, n.d.; Injury Rehabilitation Questionnaire, Deci & Ryan, n.d.) were completed.

**Results:**

Theoretical thematic analysis was conducted on three distinct phases (Early Limited Participation phase, 10 higher order themes; Late Limited Rehabilitation phase, 11 higher order themes; and Return to Play phase, 9 higher order themes) and coded relating to autonomy, competence and relatedness.

**Conclusions:**

The findings suggest that increased autonomy and control assist emotional and behavioral responses during rehabilitation and return to play, while development of competence increases self-confidence.

## Background

Injury rates have increased significantly in rugby union over the last decade [1]. The incidence of injury in the professional game has been found to be 218 and 6.1 injuries per 1000 h of competitive performance and practice respectively. Of these injuries 60% were to the lower limb and 41% were joint or ligament injuries that required on average 17 days rehabilitation [2]. In addition to this Brooks et al. [2] identified injury to the anterior cruciate ligament (ACL) as the most severe requiring an average 235 days rehabilitation. A range of negative emotions, principally shock, disbelief, anger, frustration and depression, have been reported by athletes following ACL injury [3]. Over time these negative emotions are replaced by optimism and focus as the athlete concentrates on the rehabilitation process [4]. The quicker an athlete becomes disengaged from these negative emotions the sooner they can become focused on the physical rehabilitation resulting in a speedier and more efficient return to previous physical fitness levels [5].

Cognitive appraisal models [6, 7] and stage models [8, 9] have both attempted to assist understanding of the psychological processes experienced as a consequence of sports injury. However, Podlog and Eklund ([10]: p542) suggest that self-determination theory (SDT) offers “a comprehensive perspective on the salient issues facing athletes returning to sport from injury”. This view is supported by Arden, Taylor, Feller and Webster ([11]: p1120) noting “the self-determination theory provides a framework within which to identify and organise the psychological factors that influence successful return to sport”. Ryan and Deci [12] state SDT is the degree to which people endorse their actions at the highest level of reflection and engage in the actions with a full sense of choice. They propose that motivational states subsist along a self-determination continuum, ranging from a motivation (i.e., lack of behavioral intention) to intrinsic motivation (i.e., engaging in an activity for personal reasons). An individual’s positioning on the continuum is defined by their needs for competence, relatedness and autonomy [12].

Two main findings have been identified relating SDT to sports injury rehabilitation [10]: (i) more intrinsically motivated rehabilitation climates will improve wellbeing and athlete health; (ii) the motivational climate experienced will influence health, wellbeing and performance in a different manner. A greater chance of successful return to competition is created when the needs for autonomy, competence and relatedness are satisfied [11], with increased self-determination being linked to more positive, adaptive coping responses and emotions [13]. Arden et al. [11] and Podlog and Eklund [13] found autonomy supportive injury rehabilitation environments promoted optimal functioning, psychological wellbeing and self-regulation.

## Methods

### Study design

Expanding on current practice within injury rehabilitation, this research utilizes a mixed methodological approach concurrent with the athlete’s rehabilitation through the physical rehabilitation process, to return to competition. The crucial aspect in justifying a mixed methodology is that the combination of methods focuses on the strengths of each single method. By using a combination of methods at various points in the research process, the researcher can build on the strength of each and minimise the weaknesses of a single method approach. A mixed-method can increase both the validity and the reliability of the data [14]. In particular the present study explored the views of professional rugby union players during the early rehabilitation, late rehabilitation and return to play stages, following ACL injury.

### Participants

Five professional rugby union players, who had suffered ACL injury that required surgical intervention committed to this study. Their age range was between 18 and 27 years, and the total time in rehabilitation was between 6 and 12 months. All players were playing in top tier professional rugby teams. See Table 1 for participant demographics.

**Table 1:** Participant demographics

Player	Length of time prior to surgery	Length of rehabilitation	Suffered previous severe injury
1	4 weeks	11 months	Yes
2	3 weeks	6 months	Yes
3	2 weeks	8 months	No
4	2 weeks	12 months	No
5	4 weeks	12 months	Yes

### Procedure

Initial contact was made with each player prior to them undergoing reconstructive surgery and within 2 weeks of the ACL injury being diagnosed. Following institutional ethics approval guidelines, each player completed informed consent at this initial contact time. The methodological procedure was split into three distinct phases of the injury and subsequent rehabilitation, expanding on research conducted by Shelley [15], to cover (i) Early Limited Participation – detailing the early part of the rehabilitation program, where the emphasis is on regaining the full range of movement in the knee joint and muscle strength; (ii) Late Limited Participation – detailing the final part of the rehabilitation program, where emphasis is on more sport specific training and the final preparation for full fitness; (iii) Return to Play – detailing the first three games of competition after full rehabilitation.

A mixed methodological approach was undertaken concurrent with each player’s rehabilitation, comprising of semi-structured interviews, a self-report diary and completing three established questionnaires related to SDT and social support. This approach consisted of a dominant (qualitative) – less dominant (quantitative) design as suggested by Tashakkori and Teddle [16]. The semi-structured interview guides, as utilized by Podlog and Eklund [17] focused on the player’s cognitions, emotions and coping strategies experienced during the rehabilitation, as well as the perceived control and support provided to them, and were based on previous literature (e.g., [18, 19]). Twice monthly interviews, utilizing the semi-structured guides and lasting on average 30–45 min in duration, were conducted with each player, at his club training facility, concurrent with his rehabilitation (totalling 8–16 interviews, dependent on the length of the rehabilitation process. See Table 2 for individual player specifics). In addition, each player was asked to complete a pre-designed, self-report diary to allow them to record day-to-day changes related to their emotions and coping strategies. Specifically, each player was instructed to record both positive and negative emotional changes, and indicate the strategies utilized to cope during the rehabilitation related to both the injury and life in general. Diaries allow for a more extensive investigation [20] as they can reduce the time between the event and recall. The use of personal documents, such as diaries, also enables the participant to provide detailed information relating to them personally that they may be unwilling to discuss in other forums [21]. Furthermore, three established questionnaires were completed following successful return to competition to ascertain the social support and perceived autonomy-support provided during rehabilitation. These were the MOS Social Support Survey (MOS-SSS [22]), which enabled the identification of various forms of social support (Emotional/Informational support; Tangible support; Affectionate support; Positive Social Interaction) offered during the rehabilitation, and two questionnaires adapted from the Sport Climate Questionnaire (part of a group of questionnaires used to identify Perceived Autonomy-Supportive Climates [23]). These questionnaires are designed to assess to what degree the climate established is autonomous or controlling. The final two questionnaires investigated the climate established by the coach (Sport Climate Questionnaire) and by the physiotherapist (Injury Rehabilitation Questionnaire; which replaced the word ‘coach’ with ‘physiotherapist’) during the rehabilitation process. Although there a number of other scales which assess social support, the present study included the MOS-SSS because of its good psychometric properties (Internal consistency was high for tangible support (α = .91), emotion/information support (α = .96), affection support (α = .94), positive social interaction (α = .94), and total support (α = .93) [22]), the multidimensional assessment of social support, it has been used previously in injury rehabilitation, and because it is relatively quick to complete. Perceived Autonomy-Supportive Climates questionnaires have been successfully utilized in a wide range of settings and the alpha coefficient of internal validity has been above .90 for this instrument [23].

**Table 2:** Number of interviews per player in each phase

Player	Early limited participation	Late limited participation	Return to play
1	4	4	3
2	3	2	3
3	3	3	3
4	6	4	3
5	8	5	3

### Data analysis

Each interview was audiotaped and transcribed verbatim and a theoretical thematic analysis, as suggested by Braun and Clarke [24] was conducted on the transcripts and diary entries. A theoretical thematic analysis allowed for a more specific examination of the athlete’s views in relation to SDT frameworks (autonomy; competence; relatedness). Specifically, utilizing the guidelines by Braun and Clarke, the following five stages of analysis were completed, for each phase of the rehabilitation: (i) All interviews and diary entries were read and reread to allow the analyzers to obtain familiarity with them; (ii) Significant statements and phrases that directly related to autonomy, competence and relatedness were extracted and coded; (iii) These significant statements were arranged with similar terms to form raw data themes for each phase; (iv) These raw data themes were then reviewed into higher order themes, with clear and identifiable differences between the themes; (v) Ultimately, all higher order themes from each player were compared, in order to examine the common themes endured. These common themes are of the greatest generality, meaning that no links could be uncovered among these themes.

No statistical analysis was completed on the quantitative data rather it was utilized to triangulate with the qualitative data to assist with the confirmation or rejection of the themes identified. Combining data sources allows for a more holistic view of the main factors and reduces the possibility of inaccurate interpretations of the qualitative data being attained [14]. This triangulation of different data sources increased the trustworthiness [25] of the analysis conducted. Creswell [26] noted concurrent analysis of different data sources assists building a dependable rationalization for the identified themes. Credibility was further developed by prolonged engagement, member checking, peer debriefing and negative case analysis.

## Results

Thematic analysis was conducted separately on the early limited participation phase, late limited participation phase and return to play phase, and split into the general dimensions Autonomy, Competence or Relatedness. Full results from the MOS-SSS, Sport Climate Questionnaire, and Injury Rehabilitation Questionnaire are presented in Table 3. The higher the average score the greater amount of support and autonomy being perceived.

**Table 3:** Questionnaire results

	MOS-SSS					
Player	Emotional support	Tangible support	Affectionate support	Positive social interaction	Sport climate questionnaire	Injury rehabilitation questionnaire
1	4.625	4.75	5	4	5.6	7
2	4.75	5	5	5	7	7
3	4.625	5	5	5	7	6.33
4	4.125	4.75	4	4.33	5.46	6.33
5	3.75	3	5	3.67	1	7

### Early limited participation phase

**Fig. 1:**
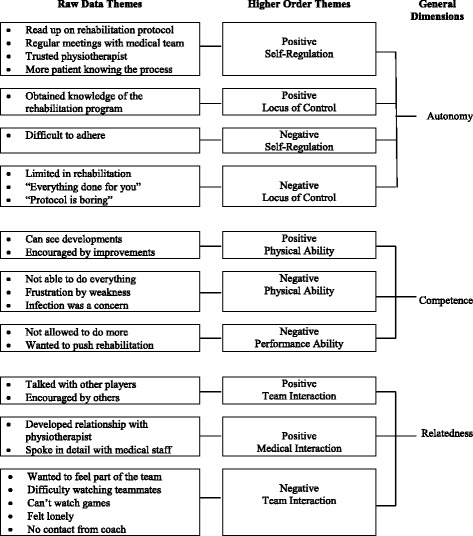
Early limited participation phase

In the early limited participation phase we identified 26 raw data themes that we combined into 10 higher order themes: *Positive Self-Regulation, Positive Locus of Control, Negative Self-Regulation, Negative Locus of Control (Autonomy)*; *Positive Physical Ability, Negative Physical Ability, Negative Performance Ability (Competence)*; *Positive Team Interaction, Positive Medical Interaction, and Negative Team Interaction (Relatedness)*. Raw data themes, higher order themes and general dimensions for this phase are presented in Fig. 1.

Of principle importance to all players was the need to understand the rehabilitation process and become personally involved in it (“Having an understanding of [the rehabilitation program] allows me to become engrossed in it” (P1); “I’m more patient knowing the process. Knowing what I can and can’t do” (P2); “I am clear on what I want to get out of the next few weeks” (P5)). The gathering of detailed information related to the rehabilitation program and increased personal control appears to be extremely beneficial to the control of emotions during this phase. The rehabilitation team can assist this process by providing a supportive setting. Each player commented on the need to trust their physiotherapist and the importance of having regular meetings with all involved in the rehabilitation (“I had regular meetings with [physiotherapist] and my fitness coach, allowing me to have some input into each session. I was involved in my rehabilitation, which was good” (P1); “I like the way [physiotherapist] allows me lots of control of what I’m doing. It’s not that I go off on my own and just do what I want, but he involves me with almost everything. It really helps to keep my interest on the job in hand and stops me from worrying about my knee and how long I’m going to be out for” (P5)). All players stated they ‘strongly agreed’ with the statements ‘*I feel understood by my physiotherapist*’ and ‘*I am able to be open with my physiotherapist*’ on the Injury Rehabilitation Questionnaire.

It is important to note that the restrictive nature of some ACL rehabilitation protocols may be debilitative for these injured players, as they limit the control each player has over the rehabilitation. Player 4 commented, “I would like some more control over what I’m doing. I lose some motivation when I’m constantly doing the same thing. The physio lets me decide on some of the exercises, but in terms of leg strengthening all the exercises are quite similar and a bit boring” and “It’s so frustrating. I feel so helpless. The rehab is just boring and repetitive”.

Seeing developments and improvements in physical ability assisted in developing competence. Player 1 stated, “I have seen a gradual improvement in my range of movement, so I am positive I am improving all the time”. Similar comments were made by all players as they reach their rehabilitation goals, with each observed improvement appearing to have a cyclical effect by increasing self-confidence which in turn increased each player’s commitment to the rehabilitation program. However, frustration can become a factor when players are unable to reach the set targets (“It’s hard to see the gap between now and when I get to play again being bridged, so it’s frustrating with lack of mobility and poor strength of my knee” (P2); “Although it’s good to be doing some activity again, it’s frustrating not being able to do as much as I want” (P4)). The impact of set ACL rehabilitation protocols may again have an impact at this early stage of rehabilitation. In many cases these protocols are highly structured stating weekly training targets, allowing little flexibility for individual performers (“I’m so bored at the moment. All I do is go training in the gym or the pool, have a bit of physio and that’s it. I just want to get on and do something exciting” (P1)).

Social support was important for each player during this phase, in particular developing a connection with their physiotherapist. Scores from the Injury Rehabilitation Questionnaire highlight the good relationship each player gained with their physiotherapist, with average scores ranging from 6.33 to 7 out of a maximum of seven. Player 5 commented, “[physiotherapist] was really good. He answered all my questions and gave me information that I could understand”. A vast amount of positive social support was received from teammates, which predominantly involved encouragement and relatedness (“the boys were always encouraging and helping me” (P1)). However, some aspects of teammate support had a negative impact on the rehabilitating player’s emotions. Comments from players included, “My only negative is having to watch the other guys training outside. I still want to be part of the squad but it’s hard seeing it from the gym all the time” (P1) and “It’s a bit lonely really. It’s not like when you train with the boys, because most of the time I’m doing things on my own” (P2). Player 5 had a number of difficulties with the lack of support provided to him by his coach (“I haven’t heard much from the coach. It’s annoying but I wasn’t expecting to have a huge amount of contact with him. There’s some other stuff going on in the club that affects him more”). The Sport Climate Questionnaire was scored at the lowest possible value by this player, indicating he perceived no support from his coach. Although the coach may try to keep emotional distances from players to reduce the chances of favoritism being perceived, in this case the coach appears to be more self-concerned (the coach’s contract was terminated while this player was still injured), which had a negative impact on the injured player’s emotions and behavior.

### Late limited participation phase

**Fig. 2:**
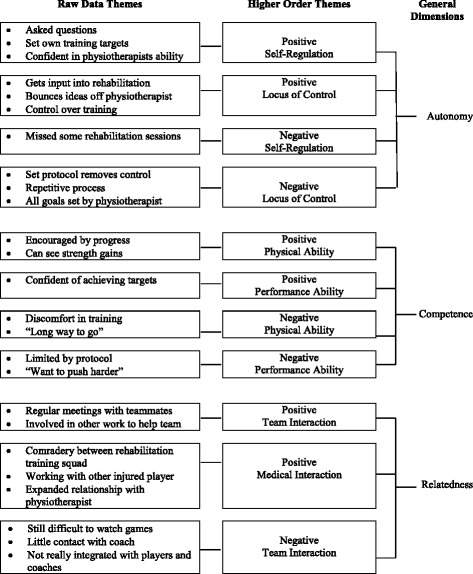
Late limited participation phase

We identified 25 raw data themes in the late limited participation phase, which we determined into the higher order themes: *Positive Self-Regulation, Positive Locus of Control, Negative Self-Regulation,* and *Negative Locus of Control* forming the general dimension *Autonomy*; *Positive Physical Ability, Positive Performance Ability, Negative Physical Ability,* and *Negative Performance Ability* comprising the general dimension *Competence;* and *Positive Team Interaction, Positive Medical Interaction, and Negative Team Interaction* forming *Relatedness*. Raw data themes, higher order themes and general dimensions for this phase are presented in Fig. 2.

Increased autonomy in the rehabilitation was experienced by all players during the late limited participation phase, with elevations in both self-regulation and locus of control (“At times in the early stages it could get very repetitive and sometimes boring, but as it gets closer and closer to the pitch it motivates me more” (P3); “I’ve suffered what I thought was a pretty big setback … I got some reassurance from the medical team. I was quite relieved. Now I am back on track, trying to do everything I can to help myself” (P4)). Again asking questions and bouncing ideas off the physiotherapist proved important to the rehabilitation. Player 3 noted, “I’m learning more and more about the injury and the best methods to rehabilitate. The medical team probably hate it because I’m asking so many questions”. This sense of control was further enhanced by the players having some input into the rehabilitation program. Comments from players included, “The step up in training is great and allowed for some variety. I got to choose whether I was on the bike or in the pool or whatever” (P1) and “I set some of my own training targets. Obviously the training staff oversees these and ensure they meet my needs, but I’m involved” (P2).

Players 4 and 5 had a slightly different experience stating, “All [goals] set by the physio. I do have some input into the different exercises. Well that’s what they say, because I don’t think they take it into account” and “There’s no need for me to be involved in goal setting. [The physiotherapist] is a specialist and I trust him”. Although the lack of autonomy experienced by these players did not appear to negatively affect their emotions, both players spent the longest time in rehabilitation.

Again competence was developed by seeing progressions in the rehabilitation and an increased confidence in the ability to achieve targets. The players in the present study with lower self-confidence stated they missed some training sessions or “didn’t give 100%”, while those with increased self-competence reported wanting to push themselves further (“I’ve done a few things that my physio shouldn’t know about to test my knee. I just needed that confidence in my own ability” (P2)). Players did comment that they were restricted by the ACL rehabilitation protocol (“I had a target to reach each session and it didn’t matter whether I could do more or less than that. When I felt good I wasn’t supposed to push myself further, which annoyed me. I knew I could do more, wanted to, but the medical staff said no” (P4)).

Having a positive medical interaction was important for relatedness. Particularly the development of comradery between players within the rehabilitation environment was highlighted as facilitative (“Training with another player helps to keep me motivated. I don’t want to let him down” (P2); “Training as part of a rehab team is good. We constantly encourage each other and it boosts you’re confidence when one of the other guys is back playing. Although sometimes it’s a case of how long will I still be here for. Generally the support is really beneficial” (P3)). High average scores for all players on the MOS-SSS indicate the range of support available. Needing a sense of belonging encouraged three of the players to undertaken alternate work within the club (“to feel part of it again”). Player 1 became involved with video and game analysis, stating “It allowed me to feel part of the team, to be involved, even though I could not physically perform. I’m motivated by being involved, and not only does it take my mind off my knee, it allows me to help the team and even develop my own game”.

A lack of relatedness had negative emotional impact on Player 5, who struggled initially with the change of coach at the club (“I’m not really integrated with the other players and coaches. We’ve got a new coach at the club. This is a worry because I don’t know them and they don’t know me”). However, as the new coach began to recognize him as part of the team, his perspective changed (“They’ve been fantastic really. The new coaches still speak to me. One of them has been really good, always asking about me and checking to see how I’m getting on. It is nice to have that support when you’re not playing. I know some guys who have just been left in the wilderness when they’ve been injured, like the coaches don’t care but it’s been really good here”).

### Return to play phase

**Fig. 3:**
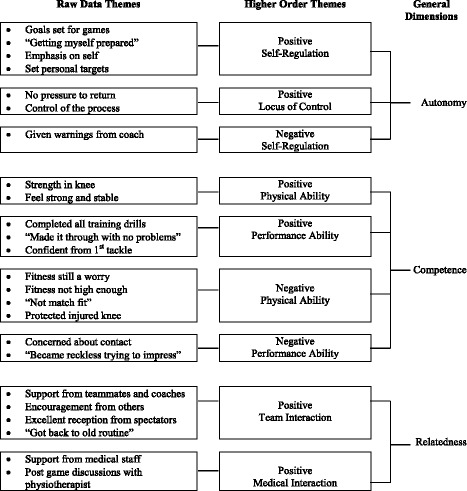
Return to play

Within the return to play phase we identified 24 raw data themes that we combined into nine higher order themes. *Autonomy* was formed by *Positive Self-Regulation, Positive Locus of Control,* and *Negative Self-Regulation*; *Competence* comprising of *Positive Physical Ability, Positive Performance Ability, Negative Physical Ability,* and *Negative Performance Ability*; and *Relatedness* including *Positive Team Interaction* and *Positive Medical Interaction*. Raw data themes, higher order themes and general dimensions for this phase are presented in Fig. 3.

A positive locus of control was experienced by all players during this phase, with none stating that they experienced any pressure to return to play (“[Coach] been good. He’s just spoken to me about being fully prepared to get back out there” (P1)). By having minimal pressure to return to play appears to greatly enhance each player’s confidence to return and reduce anxieties. All players noted the benefit of having goals set for their return (“I knew I would have a set time to play, so it meant I didn’t have to worry about lasting. I could just go full on until the end” (P2); “Having targets for my involvement meant I wasn’t concentrating on my knee, just trying to do what I needed to” (P3)). The goals set ranged from playing specific time periods to needing to be involved in play a set number of times. The only reported reduction in autonomy came from player 4 who reported being warned by his coach for trying to do too much and not concentrating on the game plan.

Competence was developed by physical and performance achievements during this phase. All players stated they had confidence in their knee (“I feel extremely ready to return. My knee feels strong and stable; and has done all through training” (P2); “I’ve been lifting some big weights in the gym … It’s just given me confidence. I know my knee’s good enough to go, so I don’t have to worry. I’d probably be shying out of tackles if I wasn’t 100%. I’ve just taken control of what I’m doing” (P1)). Greater concerns were related to a lack of physical fitness and overall performance (“I’m still nervous about how my fitness will hold up” (P1); “I am very excited about the prospect of playing a game. I’m nervous about how I will perform rather than how my knee will be” (P2)). With game time and by utilizing previous performance routines, each player was able to develop their physical competence enabling a successful return to play, at previous performance standards (“The more I played the less I focused on the knee” (P3)). Players 3 and 4 both stated they tried to protect their injured knee, by using some form of strapping or protective sleeve, and both had concerns about contact situations. This could be a predisposed trait or may be the influence of having less prior injury experience.

Relatedness within the return to play phase was positive for these players (“The coaches and the boys were excellent. The team atmosphere was good and I knew this is where I was supposed to be” (P1)). The physiotherapist was also a valuable source of support during the return to play phase. Prior to competition the physiotherapist reassured each player that their injured knee was strong, and post-game they ensured each player cared for their injured knee correctly (“I spoke to the physio and he explained that all the preseason work and training I’d done was better preparation and more rigorous than any test” (P1); “[physiotherapist] came straight up to me after the first game to check how my knee was. He told me to make sure that I iced it to stop any soreness” (P3)).

## Discussions

The aim of the present study was to explore the views of professional rugby union players during the early rehabilitation, late rehabilitation and return to play stages, following ACL injury. The discussions are presented to support and enhance critical understanding of the psychological factors associated with return to sport following ACL injury, and are provided to compliment the physical rehabilitation process. The results are discussed independently for the each of the three phases identified.

### Early limited participation phase

Within general health psychology increased personal control has led to more adaptive coping responses being utilized [27] and gathering more information about the process has facilitated rehabilitation of athletes suffering musculoskeletal injury [19] Findings from the present study supports previous research suggesting increases in self-regulation facilitate persistence [28] and adherence [29] during injury rehabilitation. Niven [30] suggests that the physiotherapist can assist in providing an autonomy supportive climate by initially establishing a relationship with the injured player and increasing the player’s confidence in both the physiotherapist and the program. Statements made by the player’s in the present study highlight the importance of gaining trust in the physiotherapist and developing a working relationship that allowed the injured player input into the rehabilitation program. Williams, Gagné, Ryan and Deci [31] noted to assist in development of autonomy each player should be allowed a considerable input to the decision making process. However, as noted previously the restrictive nature of some ACL rehabilitation protocols could limit the control each player has over the rehabilitation.

Increases in physical competence were achieved by the injured players through achievement of training targets and perceived improvement in performance. These increases in confidence could lead to better rehabilitation [32] by focusing the player on the rehabilitation goals and increasing self-confidence [33]. Players are increasingly likely to become engaged in the activity when there is a high level of self-confidence [34]. However, the present study corroborates previous research stating concerns about physical competency are common with rehabilitating athletes [4]. The present study found players to become frustrated by slow progression and considerably reduced activity levels compared to pre-injury.

Athletes with serious injury are more likely to seek social support [35] and developing a relationship with the physiotherapist may reduce the psychological trauma experienced [19]. The player’s physiotherapist became an important source during this phase, offering emotional support, information and guidance, and positive interaction. Teammates are also an important part of the social support network [36] and are most regularly available to provide support [37]. All players stated that they spoke with others who had suffered the same injury or a similar severe injury and took encouragement and inspiration from those who had successfully rehabilitated. The use of teammates as role models has been identified as facilitative to the rehabilitation process [37]. Although research suggests that it is important for injured players to maintain contact and be involved with the team, it is possible for them to have feelings of isolation [38]. Similar comments from the players in the present study highlight the frustration that can be experienced by not being around teammates [17]. To counteract this Podlog [39] recommends that coaches provide opportunities for the injured player to interact with the rest of the team.

### Late limited participation phase

Health psychology literature has identified asking questions as an active coping method, which increases perceived control of the rehabilitation process [40]. Similarly active coping has been positively correlated to improved knee function when rehabilitating following arthroscopic surgery [41]. Active coping in this manner increases autonomy during rehabilitation, with further developments obtained by allowing the player to have input into the types of activity included in the program [42, 43]. Recent research has also encouraged the health care professional to assist with this by increasing self-regulation and autonomy [38]. While the benefits of creating autonomy-supportive rehabilitation settings have been clearly acknowledged [28], and a lack of autonomy proposed to be debilitating to rehabilitation [44], players within the current study were happy to have minimal input into the program design and rather trust the qualified personnel. Further research is required to ascertain the effect of this lack of autonomy compared with high levels of trust in the health care professional, with initial propositions supporting the importance of trust to relieve anxiety and increase confidence [45].

Having realistic short-term goals can develop physical and performance competence [46], with the achievement of these being associated with: increased confidence [47]; positive emotions and mood states [11]; improved effort and commitment [38]; and higher intrinsic motivation [48]. Goal setting is a regularly utilised approach during this stage of the rehabilitation program [49] and is readily accepted by athletes, who are familiar with the process as it is typically used in training and competition. As noted in the results of the current study, the prescriptive nature of some rehabilitation protocols may have an impact on the benefits of goals setting. Adherence to the SMART goal setting principles may minimise this [50], but further research is recommended.

Relatedness is enhanced by the involvement of the injured player with significant others [30] and consistent with previous research [51] the players in this study continually sought social support. Bianco and Eklund [52] noted the need for the social support being offered to be consistent with the injured player’s needs; with the health care professional becoming more important than others during this phase [3]. Being able to maintain relationships with teammates was also crucial for the players in this study, corroborating previous findings by Clement, Granquist and Arvinen-Barrow [49] and Podlog [39]. Perceiving that there is still a connection to the team may reduce feelings of isolation [10] and act as a driver to return to competition [17]. Of interest during this research one player’s coach was replaced, which had a positive impact on the player. However the impact of this on the perceived relatedness requires further investigation.

### Return to play phase

All players in the present study stated they sensed no pressure to return and felt that they had control over the rehabilitation process, which may have facilitated a positive return experience. Gagné, Ryan and Bergmann [53] acknowledged that increased pressure and less control over the return can be detrimental, and this lack of autonomy can lead to a decrease in self-confidence and an increase in anxiety and fear of re-injury [54]. Hagger et al. [19] noted players with a greater level of perceived control report less hindrance when returning to competition, whilst Bianco [44] suggests injured players could benefit from having freedom to choose when they return because they are less likely to experience performance failures and/or re-injury. However, it is suggested that caution should be taken to ensure the player does not continually delay the return to play, resulting in exacerbated emotions. Of importance during this phase is the health care professional’s ability to create an autonomy-supportive environment [11], and particularly to ensure there is no conflict between the expectations of the coach and the returning player [51].

Physical competence is an important concern when returning to play [50] with all players required to pass clinical and sport specific tests prior to return. Successful completion of such tests is known to increase competence and self-confidence [43, 55]. Of interest is the benefit that can be ascertained by having specific targets set during the competition. While the use of goal setting has long been acknowledged to facilitate successful return from injury [56], little research has focused on the type of goals set during the actual return performance. Further research is needed but the information provided by the players in the current study corroborates recent research [45, 57] that specific performance targets minimized the fear of re-injury.

Within the literature sports medicine practitioners are encouraged to provide players returning to play with the required level of relatedness [50]. In addition to this the players in the current study gained confidence from their teammates and coaches, which may have been a result of the nature of the sport and performance level [58]. As professional rugby union players, all received an added benefit from the spectators present at their return. Further research is needed to investigate the impact of game location on the return to play, however the players who returned during a home game made more comments regarding this.

The generalization of these findings is limited by the small sample size, a characteristic that is common within qualitative research in general, however five participants is consistent with similar qualitative research in this area [3, 45]. Still it is recommended that similar research be conducted with larger numbers of participants in order to validate the higher order themes identified. This research should also be conducted concurrent to the athlete’s physical rehabilitation, as we consider this a strength of the current study. Although professional sports, in general, have high levels of medical support available the support received by these players may not correspond to that received within other organizations. Further research is suggested across a range of professional sports to understand the benefits of self-determined motivation for injured elite level performers.

## Conclusion

Developing autonomy, competence and relatedness during rehabilitation appears to facilitate effective return to competition. Self-determination can produce more adaptive coping strategies and increase adherence to the process [19]. Specifically, providing players with an in-depth understanding of the injury can increase self-regulation and provide a greater sense of control throughout the rehabilitation. Competence is developed during each phase with each player increasing physical and performance proficiency to greater extents as they progress. Meeting and achieving training targets boost confidence and players may benefit by experiencing more success throughout the early stages of rehabilitation. The physiotherapist acts as an essential source of social support to injured players, however the player’s coach may facilitate relatedness by encouraging the injured player to be involved with the team. In such ways the injured player may still feel part of the team despite not being able to physically perform.

## Abbreviations

ACL: Anterior cruciate ligament
MOSS-SSS: MOS Social Support Survey
SDT: Self-determination theory
